# Genotypic and phenotypic analysis of familial male breast cancer shows under representation of the HER2 and basal subtypes in BRCA-associated carcinomas

**DOI:** 10.1186/1471-2407-12-510

**Published:** 2012-11-09

**Authors:** Siddhartha Deb, Nicholas Jene, kConFab investigators, Stephen B Fox

**Affiliations:** 1 Department of Anatomical Pathology, Peter MacCallum Cancer Centre, East Melbourne, 3002, Australia; 2 Victorian Cancer Biobank, Victorian Cancer Council, Carlton, 3053, Australia; 3 Department of Pathology, University of Melbourne, Parkville, 3052, Australia; 4 Kathleen Cuningham Foundation Consortium for research into Familial Breast Cancer, Peter MacCallum Cancer Centre, East Melbourne, 3002, Australia

**Keywords:** Male breast cancer, BRCA1, BRCA2, BRCAX, Micropapillary, Familial

## Abstract

**Background:**

Male breast cancer (MBC) is an uncommon and relatively uncharacterised disease accounting for <1% of all breast cancers. A significant proportion occurs in families with a history of breast cancer and in particular those carrying *BRCA2* mutations. Here we describe clinicopathological features and genomic *BRCA1* and *BRCA2* mutation status in a large cohort of familial MBCs.

**Methods:**

Cases (n=60) included 3 *BRCA1* and 25 *BRCA2* mutation carries, and 32 non-*BRCA1/2* (BRCAX) carriers with strong family histories of breast cancer. The cohort was examined with respect to mutation status, clinicopathological parameters including TNM staging, grade, histological subtype and intrinsic phenotype.

**Results:**

Compared to the general population, MBC incidence was higher in all subgroups. In contrast to female breast cancer (FBC) there was greater representation of *BRCA2* tumours (41.7% vs 8.3%, p=0.0008) and underrepresentation of *BRCA1* tumours (5.0% vs 14.4%, p=0.0001). There was no correlation between mutation status and age of onset, disease specific survival (DSS) or other clincopathological factors. Comparison with sporadic MBC studies showed similar clinicopathological features. Prognostic variables affecting DSS included primary tumour size (p=0.003, HR:4.26 95%CI 1.63-11.11), age (p=0.002, HR:4.09 95%CI 1.65-10.12), lymphovascular (p=0.019, HR:3.25 95%CI 1.21-8.74) and perineural invasion (p=0.027, HR:2.82 95%CI 1.13-7.06). Unlike familial FBC, the histological subtypes seen in familial MBC were more similar to those seen in sporadic MBC with 46 (76.7%) pure invasive ductal carcinoma of no special type (IDC-NST), 2 (3.3%) invasive lobular carcinomas and 4 (6.7%) invasive papillary carcinoma. A further 8 (13.3%) IDC-NST had foci of micropapillary differentiation, with a strong trend for co-occurrence in *BRCA2* carriers (p=0.058). Most tumours were of the luminal phenotype (89.7%), with infrequent HER2 (8.6%) and basal (1.7%) phenotype tumours seen.

**Conclusion:**

MBC in *BRCA1/2* carriers and BRCAX families is different to females. Unlike FBC, a clear *BRCA1* phenotype is not seen but a possible *BRCA2* phenotype of micropapillary histological subtype is suggested. Comparison with sporadic MBCs shows this to be a high-risk population making further recruitment and investigation of this cohort of value in further understanding these uncommon tumours.

## Background

Male breast cancer (MBC) is an infrequent and poorly characterised disease. Limited data to date suggests it is epidemiologically and biologically different from female breast cancer (FBC) but it is unknown whether current paradigms and treatment of female disease can be extrapolated to the pathobiology and management of MBC and vice versa. Although some recent large MBC studies have been undertaken, these are population-based and this current report is the largest to describe the genotype, tumour phenotype, complete clinicopathological parameters and survival in MBC from high-risk families.

Accounting for less than 1% of all male cancers, and 0.65% of all breast tumours [1-3], the incidence of MBC has increased steadily from approximately 0.86 to 1.06 per 100,000 males over a 26 year period [4,5] There is controversy surrounding mortality with some suggestion that MBC disproportionately accounts for a higher number of deaths than breast cancer in women [4-7] while other studies suggest parity when comparing age and stage matched cases [8].

Inherited risk factors for MBC appears to be a more significant contributor than in women with estimates of 10% of all MBC cases arising with a family pedigree suggestive of a genetic predisposition [2,9-11]. Unlike women, *BRCA2* germline mutation in men confers a significantly higher lifetime risk of developing breast cancer than *BRCA1*[2,9-11]. Other genes also implicated in the development of MBC including *PTEN*[12], *P53*[13] and *CHEK2* 1100delC [14]. Kleinfelter’s syndrome (XXY) [15], environmental and hormonal states that alter the ratio of androgens to estrogens are also thought to contribute to MBC [16]. Recent meta-analysis has also shown an association between previous breast disease, in particular gynaecomastia, and occurrence of MBC [17]. It is still unclear, however, whether this is a; precursor lesion, a risk factor for MBC or whether the aetiology and pathogenesis is the same for both conditions.

Despite extensive knowledge about female *BRCA1*, *BRCA2* and other inherited familial breast tumours at present, little is know of male tumours from high-risk families. Comparison of sporadic tumours in both sexes shows; a steady linear increase in incidence in men with age in contrast to the bimodal distribution seen in FBC [2,3,18], an older median age of diagnosis in men [6,8,18], more advanced stage-related tumour characteristics (tumour size >2cm, positive axillary nodes) [2,18] but with more favourable histopathological characteristics (lower tumour grade) and biology (hormone receptor positive tumours) [2,18]. Most MBC studies have been performed with cohorts predominantly composed of “sporadic” population based patients whereas this study is focused on one of the largest groups of MBCs arising in high-risk families evaluating both clinicopathological and genetic associations.

## Methods

### Study group

Males with breast cancer were obtained from the kConFab repository (http://www.kconfab.org). Criteria for admission to the kConFab study has been previously published [19] (Additional file 1: Table S1) and patients were attained from within Australia and New Zealand between 1998 and 2009. The cases used in the analysis had a diagnosis of breast cancer between 1980 – 2009. Clinical parameters, including TNM staging, tumour recurrence, occurrence of non-breast primary tumours and death were obtained from referring clinical centres, kConFab questionnaires and state death registries. Information on pedigree, mutational status and testing were available from the kConFab central registry. All available slides from all cases were reviewed by a pathologist for relevant histopathological parameters. Histological classification was based on criteria set by the World Health Organisation. This work was carried out with approval from the Peter MacCallum Cancer Centre Ethics Committee (Project No: 11/61).

### Mutation detection

Mutation test results were generated through two avenues. If a clinic had performed mutation screening, the clinic report was passed onto the kConFab central registry. If no clinic mutation testing had been performed, the kConFab core research laboratory performed mutation testing. Testing for *BRCA1* and *BRCA2* mutations was performed on DNA extracted from 18 ml sample of anticoagulated blood or mouthwash kit [20]. The blood processing protocol [21] generated a nucleated cell product for DNA extraction. DNA was extracted as required (QIAamp DNA blood kit, Qiagen GmbH, Hilden, Germany). Testing of index cases in kConFab families was carried out by denaturing high performance liquid chromatography or multiplex ligation-dependent probe amplification [22]. *BRCA1* and *BRCA2* variants were classified into the following categories with criteria as posted on kConFab's website [23]: pathogenic, splice-site variant, variant of unknown significance and polymorphism. Once the family mutation had been identified, all pathogenic (including splice site) variants of *BRCA1* and *BRCA2* were genotyped by kConFab in all available family members' DNA.

### Tissue microarrays (TMAs) and expression analysis by immunohistochemistry (IHC)

TMAs were created from archival paraffin material. Two 1mm cores were taken for each tumour. TMA sections were cut at 4 μm thick intervals, de-waxed and hydrated. Antigen retrieval was performed according to manufacturers’ instructions and endogenous peroxidase activity blocked before incubating sections with desired antibodies. Tumours were separated into molecular phenotypes as per Nielsen *et al*[24]. Expression of estrogen receptor-α (ER) (Ventana, clone SP1), progesterone receptor (PgR) (Ventana, clone 1E2), epidermal growth factor receptor (EGFR) (Zymed, clone 31G7) and cytokeratin (CK) 5 (Cell Marque, clone EP1601Y) was performed. HER2 amplification was assessed by silver in situ hybridisation (SISH) using the INFORM HER2 DNA probe (Ventana). Nuclear expression of ER and PgR was scored as per the Allred scoring system [25] (intensity + percentage of tumour cells staining, 0–8) and separated into absent (score 0/8), low (1-5/8) and high (6-8/8). HER2 gene status was reported as the average number of copies of the HER2 gene per cell in 30 tumour cells. Gene status was assessed as per the guidelines recommended by Wolff *et al*[26]. EGFR was scored positive for any membranous staining of tumour cells. Expression of CK5 was defined as positive when cytoplasmic and/or membranous staining was observed in tumour cells. Tumours were assigned to the following subtypes; Luminal (ER positive, HER2 negative), HER2 (HER2 positive), Basal (ER PgR and HER2 negative, CK5 and/or EGFR positive), and Null/negative (ER, PgR, HER2, CK5/6 and EGFR negative).

### Statistical analysis

Comparison of groups was made with using Mann–Whitney U for non-parametric continuous distributions and chi-square test for threshold data. Kaplan-Meier survival curves were plotted using breast cancer related death as the endpoint and compared using a log rank test. Regression analyses as time to fail curves were plotted for age of diagnosis and occurrence of second non breast primary tumours. Cox proportional hazard regression model was used to identify independent prognostic factors for disease specific survival (DSS). Analysis was performed with GraphPad Prism 5 software (GraphPad Prism version 5.04 for Windows, GraphPad Software, La Jolla California USA). A two-tailed P-value test was used in all analyses and a P-value or less than 0.05 was considered statistically significant.

## Results

### Mutation analysis

The prevalence of MBCs in the kConFab registry with known gene mutations is summarised in Table 1 and 2. There were 5 (1.2%) of 429 known *BRCA1* mutation carriers and 35 (10.3%) of 339 *BRCA2* carriers who developed breast cancer. Of these, 3 and 25 cases respectively had reports, slides and tissues available for examination and were included in the study. Of the 3 *BRCA1* cases, 2 had a pathogenic mutation with 1 large genomic rearrangement. Of the 25 *BRCA2* cases, 22 had a pathogenic mutation, 2 large genomic rearrangements and 1 an unclassified variant. Within non-*BRCA1*/2 families, of a total of 19,137 males, 78 (0.4%) developed breast cancer with 32 cases available for use in the study.

**Table 1:** Mutation carrier status and male breast cancer with the kConFab cohort

	**BRCA1**	**BRCA2**	**Non-BRCA1/2**
All males in kConFab registry	429	339	19137
Breast Cancers	5 (1.2%)	35 (10.3%)	78 (0.4%)
Pathology Available	3	25	32

**Table 2:** **Characterisation of *BRCA1 *** **and *2 *** **mutations of males included within this study**

**Gene**	**Mutation**	**Effect**
*BRCA1*	BRCA1 del exons 21_24	LGR
	BRCA1 2798_2801 del GAAA (STOP 998)	P
	BRCA1 5382_5383 ins C (STOP 1829)	P
*BRCA2*	BRCA2 del exons 1_2	LGR
	BRCA2 del exons 14_16	LGR
	BRCA2 2988 del C (STOP 959)	P
	BRCA2 2988 del C (STOP 959)	P
	BRCA2 5873 C>A (S1882X)	P
	BRCA2 5950_5951 del CT (STOP 1909)	P
	BRCA2 5950_5951 del CT (STOP 1909)	P
	BRCA2 6024_6025 del TA (STOP 1943)	P
	BRCA2 6503_6504 del TT (STOP 2098)	P
	BRCA2 6714_6717 del ACAA (STOP 2166)	P
	BRCA2 6854_6855 del TA (STOP 2223)	P
	BRCA2 6971_6983 del ATGCCACACATTC (STOP 2275)	P
	BRCA2 698_702 del AGTCA (STOP 180)	P
	BRCA2 7708 C>T (R2494X)	P
	BRCA2 8168_8169 ins C (STOP 2661)	P
	BRCA2 9132 del C (STOP 2975).	P
	BRCA2 9161 C>A (S2978X)	P
	BRCA2 9610 C>T (R3128X)	P
	BRCA2 9610 C>T (R3128X)	P
	BRCA2 983_986 del ACAG (STOP 275)	P
	BRCA2 995 del C (STOP 276)	P
	BRCA2 del exons 1_27	P
	BRCA2 IVS 7–1 G>A	P
	P BRCA2 8525 del C (STOP 2776).	P
	BRCA2 8714 A>G (del exon 19)	UV

Classification of Variants: P = Pathogenic, LGR = Large Genomic Rearrangement, UV = Unclassified Variant.

### Clinicopathological features

**Figure 1:**
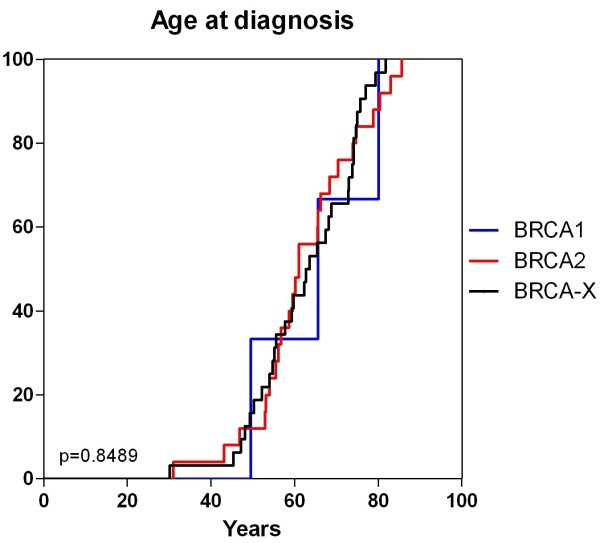
Mutation carrier status and age of diagnosis.

The clinicopathological features are summarised in Table 3. The overall median age of diagnosis was 62.5 years (range 30.1-85.6 years), and mean age of diagnosis 60.0 years. There was no significant difference in clinicopathological factors between *BRCA1*, *BRCA2* carriers and BRCAX males including age of onset (Figure 1). Surgical treatment was by wide local excision (33.3%, 20/60) and mastectomy (66.6%, 40/60). All tumours were present within 30mm of the subareolar region and the nipple. Four cases (6.6%) had multifocal disease with 2 cases of bilateral breast cancer, of which one was a metachronous BRCAX tumour with a 10 year interval and the other a *BRCA2* carrier with contralateral tumour occurring 12 years after the primary lesion.

**Table 3:** Clinicopathological features

	**All patients (n=60)**	**BRCA1 (n=3)**	**BRCA2 (n=25)**	**BRCAX (n=32)**	**P-value**
**AGE AT DIAGNOSIS**					
Median	62.5 (30.1 - 85.6)	65.6 (49.5-80.1)	61 (31.0 - 85.7)	63.2 (30.1 - 81.8)	
<60 yoa	26 (43.3%)	1 (33.3%)	11 (44.0%)	14 (43.8%)	
>60 yoa	34 (56.7%)	2 (66.6%)	14 (56.0%)	18 (56.3%)	NS
**DISEASE SPECIFIC MORTALITY**	35.0%	33.3%	40.0%	31.3%	
**SIDE**					
Right	36 (60.0%)	1 (33.3%)	17 (68.0%)	18 (56.2%)	
Left	24 (40.0%)	2 (66.7%)	8 (32.0%)	14 (43.8%)	NS
Unifocal	56 (93.3%)	3 (100%)	22 (88.0%)	31 (96.9%)	
Multifocal	2 (3.3%)	0	2 (8.0%)	0	
Bilateral	2 (3.3%)	0	1 (4.0%)	1 (3.1%)	NS
**HISTOLOGICAL SUBTYPE**					
Invasive Ductal Carcinoma - No special type	46 (76.7%)	2 (66.7%)	18 (72%)	28 (87.5%)	
IDC with Micropapillary component	8 (13.3%)	0	6 (24%)	2 (6.3%)	
Invasive Papillary Carcinoma	4 (6.7%)	1 (33.3%)	1 (4%)	2 (6.3%)	
Invasive Lobular Carcinoma	2 (3.3%)	0	0	2 (6.3%)	NS
**BRE GRADE**					
1	2 (3.3%)	0	1 (4%)	1 (3.1%)	
2	31 (51.7%)	0	12 (48%)	19 (59.4%)	
3	27 (45.0%)	3 (100%)	12 (48%)	12 (37.5%)	NS
**ER STATUS (ALLRED 0-8)**					
0	1 (1.7%)	0	0	1 (3.3%)	
1-5.	5 (8.6%)	1 (33.3%)	2 (8.0%)	2 (6.7%)	
6-8.	52 (89.7%)	2 (66.7%)	23 (92.0%)	27 (90.0%)	
NA	2	0	0	2	NS
**PR STATUS (ALLRED 0-8)**					
0	5 (8.8%)	0	1 (4%)	4 (13.8%)	
1-5.	8 (14.0%)	0	5 (20%)	3 (10.3%)	
6-8.	44 (77.2%)	3 (100%)	19 (76%)	22 (75.9%)	
NA	3	0	0	3	NS
**HER2**					
Amplification	5 (9.1%)	0	2 (8.3%)	3 (10.7%)	
Non-amplified	50 (90.9%)	3 (100%)	22 (91.7%)	25 (89.3%)	
NA	5	0	1	4	NS
**PHENOTYPE**					
Basal	1 (1.7%)	0	0	1 (3.3%)	
Luminal	52 (89.7%)	3 (100%)	23 (92.0%)	26 (86.7%)	
HER2	5 (8.6%)	0	2 (8.0%)	3 (10.0%)	
NA	2	0	0	2	NS
**TUMOUR SIZE**					
Median	17mm (2-50mm)	15mm (9-25mm)	17mm (6-40mm)	16 (2-50mm)	
**TUMOUR STAGE**					
T1a	1 (1.7%)	0	0	1 (3.1%)	
T1b	8 (13.3%)	1 (33.3%)	4 (16.0%)	3 (9.4%)	
T1c	31 (51.7%)	1 (33.3%)	10 (40.0%)	19 (59.4%)	
T2	19 (31.7%)	1 (33.3%)	11 (44.0%)	7 (21.9%)	
T3	1 (1.7%)	0	0	1	NS
**LYMPHOVASCULAR INVASION**					
Absent	32 (57.1%)	2 (66.7%)	14 (60.9%)	16 (53.3%)	
Present	24 (42.9%)	1 (33.3%)	9 (39.1%)	14 (46.7%)	
NA	4	0	2	2	NS
**PERINEURAL INVASION**					
Absent	31 (56.4%)	3 (100%)	12 (50.0%)	16 (57.1%)	
Present	24 (43.6%)	0	12 (50.0%)	12 (42.9%)	
NA	5	0	1	4	NS
**PAGET'S DISEASE OF NIPPLE**					
Absent	44 (84.6%)	2 (100%)	19 (86.4%)	23 (82.1%)	
Present	8 (15.4%)	0	3 (13.6%)	5 (17.9%)	
NA	8	1	3	4	NS
**NODAL STATUS**					
Cases with nodes examined	46 (76.7%)	3 (100%)	20 (80.0%)	23 (71.9%)	
Cases with positive nodes	20 (43.4%)	2 (66.7%)	9 (45.0%)	9 (39.1%)	NS
Average numbers of nodes examined per case	12.9 (1-30)	16.3 (13-24)	15.9 (1-30)	10.1 (1-29)	
NODAL STAGE					
N0	26 (56.5%)	1 (33.3%)	11 (55.0%)	14 (60.1%)	
N1	18 (39.1%)	2 (66.7%)	8 (40.0%)	8 (34.8%)	
N2	2 (4.3%)	0	1 (5.0%)	1 (4.3%)	NS
Cases with extranodal extension	8 (17.4%)	0	5 (25.0%)	3 (13.0%)	NS
**MARGINS**					
Clear	29 (48.3%)	1 (33.3%)	12 (48.0%)	16 (50.0%)	
Involved	15 (25.0%)	0	6 (24.0%)	9 (28.1%)	
Not assessable	16 (26.7%)	2 (66.7%)	7 (28.0%)	7 (21.9%)	NS
**DCIS**					
Absent	14 (25.0%)	0	7 (29.2%)	7 (24.1%)	
NA	4	0	1	3	
Present	42 (75.0%)	3 (100%)	17 (70.8%)	22 (75.9%)	NS
Nuclear Grade					
Low	2 (4.8%)	0	2 (11.8%)	0	
Intermediate	26 (61.9%)	1 (33.3%)	10 (58.8%)	15 (68.0%)	
High	14 (33.3%)	2 (66.7%)	5 (29.4%)	7 (31.8%)	NS

NS – Not significant.

**Figure 2:**
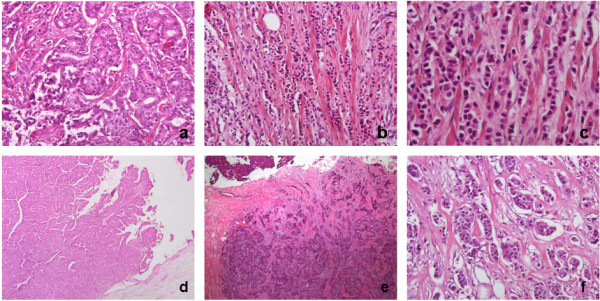
H&E histological subtypes in male breast cancer: a) invasive ductal carcinoma of no special type, b) & c) invasive lobular carcinoma, d) & e) invasive papillary carcinoma, f) invasive micropapillary carcinoma.

Tumour size ranged from 2 mm to 50 mm (median 17 mm). The most common histological subtype was infiltrating ductal carcinoma of no special type (IDC-NST) (90%, 54/60) (Figure 2a) with 2 cases of invasive lobular carcinoma (3.3%) (Figure 2b and c) and 4 cases of invasive papillary carcinoma (6.7%) (Figure 2d and e). Of the IDC-NST tumours, 8 had areas between 15 to 40% of invasive micropapillary carcinoma (Figure 2f).

Tumours were of mainly grade 2 (51.7%) and grade 3 (45.0%). Lymphovascular and perineural invasion (PNI) was identified in 42.9% (24/56) and 43.6% (24/55) of cases respectively when able to be assessed. Paget’s involvement of the nipple was seen in 15.4% of cases (8/52) when assessable. Most tumours had a component of DCIS present (75%, 42/56). Normal breast tissue and gynaecomastia was observed in 65.1% (28/43) and 11.6% (5/43) of cases respectively. Forty six cases had lymph node sampling with 7 sentinel node biopsy only (15.2%) and the remainder axillary dissection (84.7%). On average 1.6 sentinel nodes (median 1, range 1–3) were examined and an average of 15 nodes from axillary dissections (median 13, range 4–30). Of these, 1 (14.3%) sentinel node had metastatic disease and 19 axillary dissections had positive nodal disease (48.7%) with extranodal extension in 8 cases.

**Figure 3:**
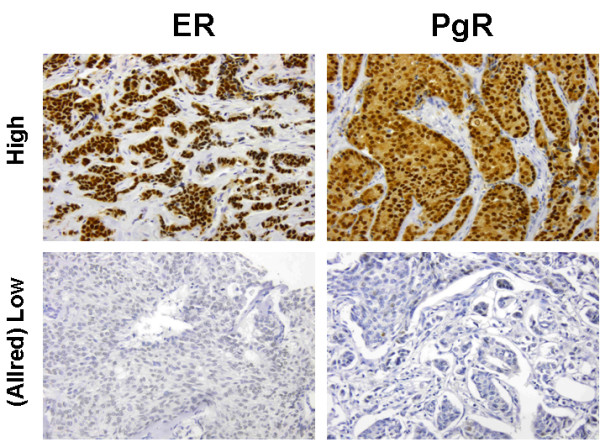
Immunohistochemical staining of male breast cancer for ER and PgR.

**Figure 4:**
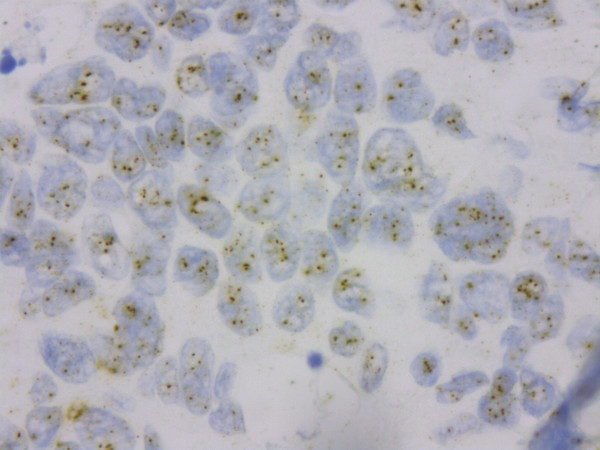
HER2 SISH demonstrating HER2 amplification in male breast cancer.

**Figure 5:**
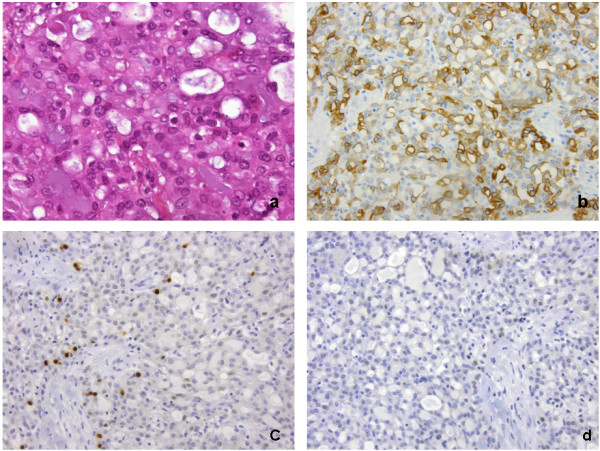
Male breast cancer of basal cell phenotype: a) H&E, b) CK5, c) ER, d) PgR.

Most tumours were ER and PgR positive (Additional file 2: Figures S1 and Additional file 3: Figure S2), with 89.7% (52/58) and 77.2% (44/57) of cases respectively scored as high (Allred score 6-8/8) (Figure 3). HER2 amplification was seen in 9.1% (5/55) of cases (Figure 4). The range of HER2 amplification was 6.1-10.5 signals per nuclei in amplified cases. Two tumours were unable to be immunophenotyped completely. Based on analysis of the remainder, the most common intrinsic subtype was Luminal (89.7%, 52/58) followed by HER2 (8.6%, 5/58) and Basal (1.7%, 1/58). The Basal subtype (Figure 5) was a BRCAX tumour with prominent CK5 and EGFR staining but also low ER nuclear positivity. Morphology of this tumour was more consistent with a basal subtype rather than a luminal type tumour.

There was a trend towards *BRCA2* tumours having an invasive micropapillary component (24% 6/25, p=0.0574) and high Bloom Richardson Ellis (BRE) grade for *BRCA1* tumours (100% grade 3 3/3, p=0.0855), however these observations did not reach statistical significance. Overall, clinicopathological factors and intrinsic subtypes were not associated with *BRCA1* or *2* mutation carrier status and unlike in female breast cancer [27], there was no association between *BRCA1* mutational status and basal cell phenotype.

Characteristics are compared with other recent large MBC studies containing >50 patients and completed within the last 4 years [6-8,28-40] (Additional file 4: Table S2) and with the previous study of female breast cancers within the kConFab cohort [41].

### Disease specific survival

**Figure 6:**
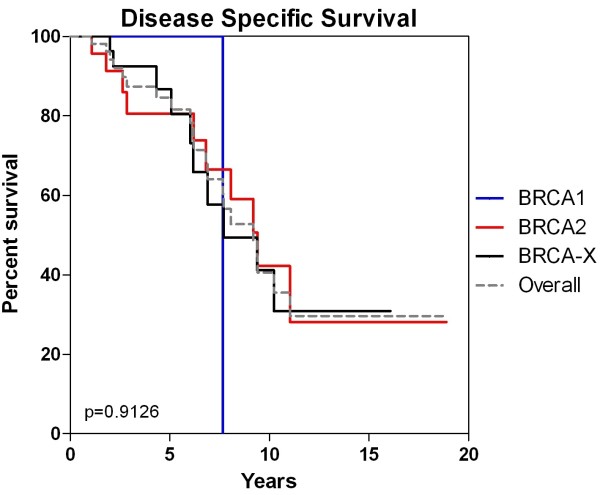
Mutation carrier status and disease specific survival.

**Figure 7:**
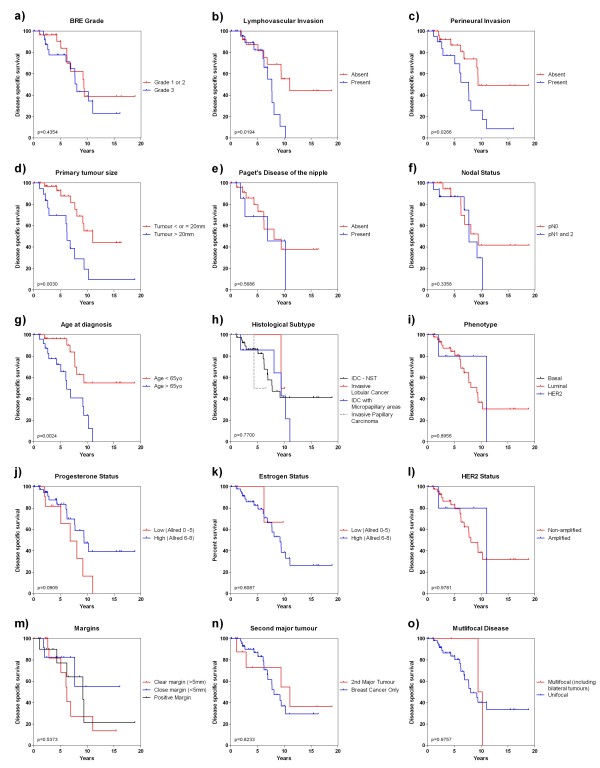
Clinicopathological variables and disease specific survival: (a) BRE grade, (b) lymphovascular invasion, (c) perineural invasion, (d) primary tumour size, (e) Paget’s disease of the nipple, (f) nodal status, (g) age at diagnosis, (h) histological subtype, (i) Intrinsic phenotype, (j) PgR immunohistochemical expression, (k) ER immunohistochemical expression, (l) HER2 amplification, (m) involvement of margins, (n) diagnosis of second non breast primary malignancy, (o) multifocal disease.

The overall 5 and 10 year disease specific survival rates were 84.6% and 40.6% for all cases, 100% and 0% for *BRCA1* case, 80.6% and 42.2% for *BRCA2* cases and 86.7% and 41.2% for BRCAX cases (Figure 6). Clinicopathological variables (Figure 7) that were of prognostic significance for DSS included a primary tumour size >2.0 cm (HR:4.26 95%CI 1.63-11.11, p=0.003), age at diagnosis > 65 years (HR:4.09 95%CI 1.65 -10.12, p=0.002), lymphovascular invasion (HR:3.25 95%CI 1.21-8.74, p=0.019) and PNI (HR:2.82 95% CI 1.13-7.06, p=0.027) (Table 4). A strong adverse trend for loss or low progesterone receptor expression was also seen (HR:2.59 95%CI 0.86-7.80, p=0.091) but fell short of being statistically significance.

**Table 4:** Clinicopathological variables of prognostic significance

**Variable**	**P-value**	**Hazard's ratio**	**95% confidence interval**
Lymphovascular Invasion	0.0194	3.25	1.21 - 8.74
Perineural Invasion	0.0266	2.82	1.13 - 7.06
Tumour Size > 20mm	0.0030	4.26	1.63 - 11.11
Age of Diagnosis > 65 years	0.0024	4.09	1.65 - 10.12
Low Progesterone Receptor Expression	0.0909	2.59	0.86 - 7.80

Comparisons of mutation carrier status, tumour grade, presence of nodal disease, involvement of surgical margins and multifocality were not prognositically significant (all p>0.05).

### Second cancers

**Figure 8:**
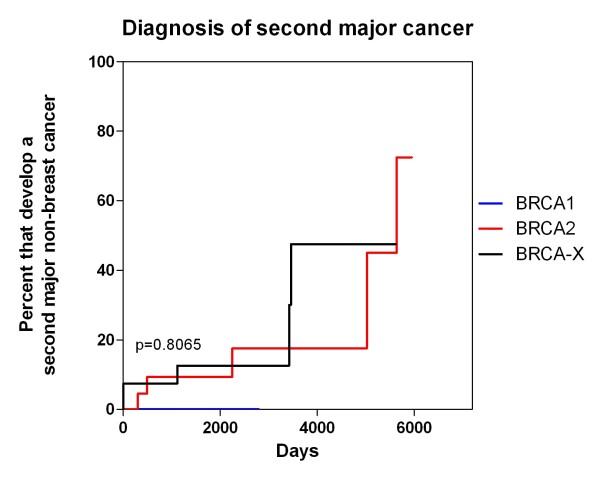
***BRCA *** **status and onset of second malignancy.**

Ten patients had a second major malignancy (5/25 *BRCA2* mutation carriers, 5/31 BRCAX cases) (Table 5). No *BRCA1* patients developed a second malignancy. In eight (80%) cases, the diagnosis of the primary breast tumour was the sentinel event while in two cases (20%) another malignancy was diagnosed preceding the breast cancer. The median time to diagnosis was 3.8 years after the diagnosis of the breast cancer (range 3 years previous to 15.5 years after). The most common second malignancy was prostatic acinar adenocarinoma (50%, 5/10). Of note, one patient had an adenocarcinoma of the abdominal wall of unknown primary origin with exclusion of a breast metastasis. Mutation carrier status was not prognostic of development of a second malignancy when comparing *BRCA2* and BRCAX cohorts (Figure 8).

**Table 5:** Second non-breast primary malignancies

**Gene**	**Mutation**	**2nd tumour**	**Diagnosis relative to Breast Primary (years)**
*BRCA2*	BRCA2 2988 del C (STOP 959)	Ascending Colon - Adenocarcinoma	15.5
*BRCA2*	BRCA2 698_702 del AGTCA (STOP 180)	Prostate - Acinar Adenocarcinoma	0.8
*BRCA2*	BRCA2 8168_8169 ins C (STOP 2661)	Prostate - Acinar Adenocarcinoma	1.4
*BRCA2*	BRCA2 del exons 1_27	Parotid gland - Oxyphilic adenocarcinoma	6.2
*BRCA2*	BRCA2 IVS 7–1 G>A	Lung - squamous cell carcinoma	13.8
BRCAX		Adenocarcinoma - unknown primary	3.0
BRCAX		Lung – Carcinoma not otherwise specified.	9.4
BRCAX		Prostate - Acinar Adenocarcinoma	9.5
BRCAX		Prostate - Acinar Adenocarcinoma	3.0 years prior to breast cancer
BRCAX		Prostate - Acinar Adenocarcinoma	1.3 years prior to breast cancer

## Discussion

To the best of our knowledge this is the largest high-risk population based study to date describing the genotypic, conventional clinicopathological and intrinsic phenotypic characteristics of MBCs arising within breast cancer families. Previous studies have either not contained large numbers of patients with a significant family history [30,34,35,37,43,47], not commented or examined family history [6-8,28,29,36,39,40], or have contained large numbers of such cases with strong family pedigree but not described clinicopathological features [32] (Table 4). As a large proportion of MBCs are purported to arise in families with breast cancer and in particular *BRCA2* mutation carriers, further description of this cohort is of significance in understanding and characterising the disease.

The incidence of MBC in *BRCA2*, *BRCA1* and BRCAX males is significantly higher than the lifetime cumulative incidence of 0.1% in the general population [17,48] confirming this group as a high risk for MBC. However, the representation of carriers is different to that of familial FBC with direct comparison within the kConFab registry [41] showing an increased proportion of *BRCA2* male carriers and underrepresentation of *BRCA1* male tumours. This suggests that significant gender associated modifiers such as high estrogen levels may affect *BRCA1* penetrance over *BRCA2*. Comparing studies of sporadic MBC [6-8,28-32,35,37-40,44], the median and mean age of onset in our patients is also younger, and this together with the observation of frequent multifocality or bilateral disease reflects the pattern of cancer often seen with underlying genetic predisposition as seen in familial FBC. A recent large population based study by Ottini *et al. *[45] containing 46 *BRCA2* mutation carriers also observed a high rate (15.2%) of contralateral breast cancer in these carriers, thus supporting this observed pattern.

Compared with other MBC groups, our study appeared to have a higher proportion of high grade tumours with only 3.3% of tumours of BRE grade I, the lowest within any MBC cohort reported to date. We also reported the highest proportion of invasive papillary carcinomas with 6.7% of cases, the next highest in the literature being 5.5% by Ottini *et al.*[45]. The histopathological tumour characteristics of our group otherwise is comparable to that seen in previous studies of sporadic MBC with the majority of cancers being invasive ductal carcinoma. This is higher than that seen in FBCs from kConFab [41]. Unlike FBC, we also observed proportionately less lobular carcinoma which is thought to reflect paucity of lobular and acinar units in males [49].

We also report a relatively higher proportion of tumours with invasive micropapillary areas particularly within *BRCA2*-associated tumours, an association not previously reported. Recent studies suggest that these lesions are a distinct entity with more aggressive behaviour than IDC-NST [50]. The distinct histological features of these tumours correlate with distinct molecular genetic profiles [42], however, in female cancer a correlation with *BRCA2* mutation has not been described or suggested [10]. Ottini *et al*[45], also describe a *BRCA2* MBC phenotype with a high proportion of BRE grade 3 tumours (54.8%), loss of PgR expression (67.9%) and HER2 amplification (63.2%). Similar to them, our *BRCA2* carriers contained a large proportion of BRE grade 3 but was not significantly different to the *BRCA1* and BRCAX population. The expression of ER and PgR in our familial MBCs is similar to that seen in sporadic MBC, with proportionately higher levels than seen in FBC, and absence of PgR expression did not discriminate a *BRCA2* phenotype. Subsequently, the majority of our cases were also of the luminal subtype. Reported HER2 amplification in MBC has been more variable than ER and PgR with studies demonstrating between 3.3% [40] to 28.4% [45] of cases showing HER2 amplification. While our study and Ottini are the only to date to examine the association with *BRCA* status, using routine diagnostic testing for HER2 we see lower frequency of HER2 amplification both overall (9.1%) and within our *BRCA2* carriers (8.3%) as a subgroup. Our results are consistent with most MBC studies that suggest HER2 amplification is seen half as frequently as that in FBC [41].

The few numbers of *BRCA1* MBCs in our cohort precludes extensive clinicopathological analysis, however, in contrast and unlike tumours seen in *BRCA1* female carriers [27,51], cancers of medullary/basal cell phenotype in *BRCA1* males has not been reported in the literature and was also not observed in our cohort of *BRCA1* males. The paucity of tumours of basal phenotype in our cohort overall also reflected observations of other MBC studies.

Several prognostic markers in our study are also reported in both FBC and sporadic MBC. In our study, we confirmed many but also identified PNI as being of prognostic significance, which has not been reported previously in MBC. Its presence, being double most rates reported in FBC [52,53], may be due to frequent subareolar tumour location which is less frequently seen in women, and comparable to frequent perineural involvement seen in other epithelial tumours such as pancreatic [54] and prostatic [55] adenocarcinoma where the organs have closer proximity to nerve bundles. While mixed prognostic significance of PNI has been seen in FBC studies [53], PNI positive tumours have been shown to be more often associated with positive nodal status and hormonal positivity [53], both of which are more commonly seen in MBC in general, and in our study cohort when compared with FBC.

While our numbers are not large, a considerable proportion (16.6%) of the *BRCA2* and BRCAX patients developed a second non-breast primary malignancy. The onset or histological type of these tumours did not correlate with mutation carrier status. These findings are consistent with those previously reported in MBC cohorts where the range of second cancer incidence varies between 5.9% to 22.8% when reported [8,28,30,31,34,35]. Notably, the studies with higher rates of breast cancer families such as Ding [31] (60% either *BRCA2* pathogenic mutation carrier or strong family history of breast cancer), Liukkonen[35] (33.1% with significant familial history) and Kiluk [34] (29% with significant familial history) had 22.8%, 19% and 19.4% of their patients reporting a second primary respectively. Of the types reported, prostate cancer was the most common followed by bladder cancer, a tumour type not seen in our cohort. In recent studies we and others have demonstrated the relative risk for developing prostate cancer in male *BRCA2* mutation carriers as between 2.9 to 4.8 times the general population [56-59]. Comparing our study with the age related rate of Australian males in the 60–64 year age group, there is an increased relative risk of prostate cancer of 19.08 (p<0.0001, 95%CI 4.50-80.91) and 20.56 (p<0.0001, 95%CI 6.30-67.12) times the normal population for *BRCA2* and BRCAX male patients with breast cancer respectively. These data show that patients with MBC may be a high-risk group for developing second malignancies, even when comparing with BRCA2 carriers without MBC. Whether this is due to hormonal influence driving both tumour types or underlying genetic factors requires further study in a larger data set.

## Conclusions

This is the largest clinicopathological study of male breast cancers arising in breast cancer families. It identifies three high-risk population groups (*BRCA1*/2, BRCAX) which may be important for screening for male breast cancer. The clinical and pathological characteristics are different to familial female breast cancer but similar to previously described male breast cancer studies which have contained but not separately analysed sporadic and familial breast cancers. Notably, our study in comparison contains proportionately more multifocal disease, a younger age of onset and a significant proportion with a second major malignancy, features often seen in tumours that arise with a genetic predisposition. *BRCA*2 mutation status did not appear to correlate with a distinct clinicopathological phenotype or disease behaviour, and a strong trend was seen within *BRCA2* carrier tumours containing areas of micropapillary carcinoma possible suggesting a possible *BRCA2* male breast cancer phenotype. Further subgroup analysis, in particular of *BRCA1* tumours, was limited by the number of cases available. Further recruitment of well characterised tumours in breast cancer families, in particular a focused collection of *BRCA1* cases, is warranted to validate and characterise familial MBC further.

## Abbreviations

MBC: Male Breast Cancer; FBC: Female Breast Cancer; DSS: Disease Specific Survival; ER: Estrogen Receptor; PgR: Progesterone Receptor; CK: Cytokeratin; EGFR: Epidermal Growth Factor Receptor; IDC-NST: Invasive Ductal Carcinoma of No Special Type; PNI: Perineural Invasion; BRE: Bloom Richardson Ellis.

## Competing interests

The authors declare they have no competing interests.

## Authors’ contributions

SD – Pathology review of all cases, conception and design of study, analysis and interpretation of data, statistical analysis, manuscript preparation, NJ – Immunohistochemistry and ISH, kConFab Investigators – *BRCA1/2* testing, acquisition of all data, manuscript preparation, SBF – Conception and design of study, analysis and interpretation of data, manuscript preparation. All authors read and approved the final manuscipt.

## Pre-publication history

The pre-publication history for this paper can be accessed here:

http://www.biomedcentral.com/1471-2407/12/510/prepub

## Supplementary Material

Additional file 1 **Table S1.** Eligibility criteria for recruitment of families into kConFab. Click here for file

Additional file 2 **Figure S1.** Distribution of ER Allred histoscores. Click here for file

Additional file 3 **Figure S2.** Distribution of PgR Allred histoscores. Click here for file

Additional file 4 **Table 2.** Comparison of previous MBC studies. Click here for file
