# Supplementary material for: Genotypic and phenotypic analysis of familial male breast cancer shows under representation of the HER2 and basal subtypes in BRCA-associated carcinomas
Source: BMC Cancer. 2012 Nov 9;12:510. doi: 10.1186/1471-2407-12-510 (PMC3561656; doi:10.1186/1471-2407-12-510)
Supplement: Additional file 1 — Table S1. Eligibility criteria for recruitment of families into kConFab. [file 1471-2407-12-510-S1.doc]

Supplementary Table 1: Eligibility criteria for recruitment of families into kConFab.

| **CATEGORY 1. Families in which no predisposition mutation has been identified.** All criteria are required: | At least one member of the family at high risk according to the National Breast Cancer Centre Category 111 guidelines (www.nbcc.org.au) |
| --- | --- |
|  | Four or more cases of breast or ovarian cancer (on one side of the family) |
|  | Two or more living affected with breast or ovarian cancer |
| **CATEGORY 2. Families in which a BRCA1 or BRCA2 mutation has been identified (pathogenic, splice site or unclassified variant).** kConFab will recruit all families in which there are at least two or more living potential female mutation carriers (affected and/or unaffected) amongst first and second degree relatives from the informative side of the family. | Mutation status of potential carries may be unproven, but simply predicted by Mendelian inheritance |
|  | There does not need to be a living affected potential carrier |
|  | One or both potential carriers can be unaffected |
| **CATEGORY 3. Families with mutations in other breast cancer predisposition genes.** A small number of pedigrees submitted by the FCC have some features of other cancer syndromes that include breast cancer and are of interest to kConFab because they carry mutations in PTEN, TP53 or ATM. Families that carry pathogenic mutations in these genes and have two or more living carriers, or potential carriers are eligible for enrolment into kConFab. Families must carry a mutation to be enrolled into Category 3. *If the clinical features suggest a relevant cancer syndrome (eg LiFraumeni Syndrome) but no mutation has been identified, the family can only be enrolled if they fulfil the Category 1 criteria.* |  |
| **CATEGORY 4. High risk breast cancer families from which tumour is available but who do not fit other kConFab criteria.** Families that fit the National Breast Cancer Centre Category 111 guidelines but do not fit category 1, 2 or 3 above are of value to kConFab if a family member wishes to enrol in kConFab and consent for a portion of their tumour (breast and ovarian) to be collected from surgery and used for research. | Families are recruited through category 4 if a woman is having surgery for a suspected tumour. Normal tissue is collected that is prophylactically removed at the time of this surgery for a suspected tumour |
|  | Prophylactic mastectomies are collected from women already enrolled in kConFab |
|  | Prophylactic oophorectomies have been previously collected but no longer currently. |
