# Supplementary figures and images for: Genotypic and phenotypic analysis of familial male breast cancer shows under representation of the HER2 and basal subtypes in BRCA-associated carcinomas
Source: BMC Cancer. 2012 Nov 9;12:510. doi: 10.1186/1471-2407-12-510 (PMC3561656; doi:10.1186/1471-2407-12-510)

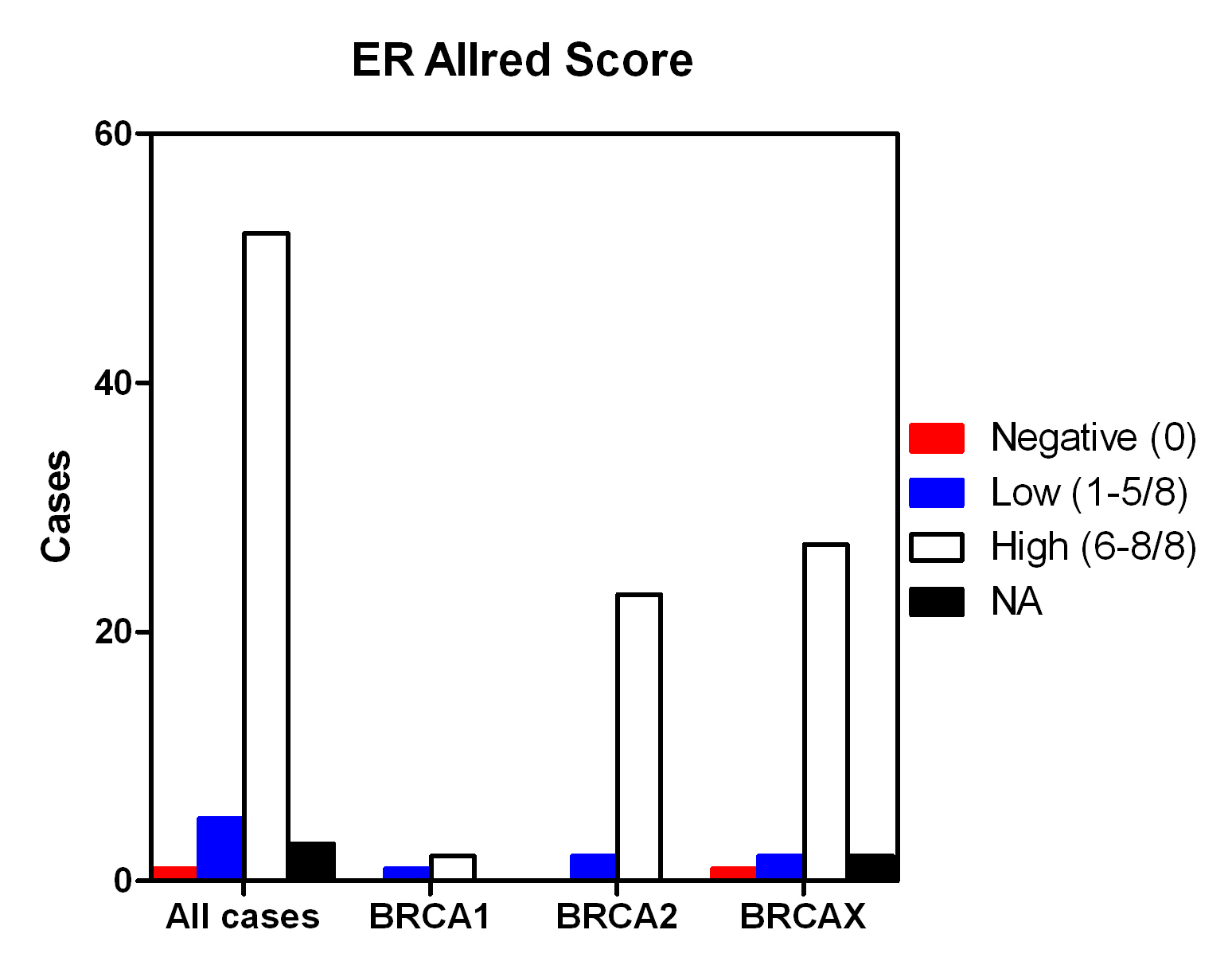

Supplement: Additional file 2 — Figure S1. Distribution of ER Allred histoscores. [file 1471-2407-12-510-S2.jpeg]

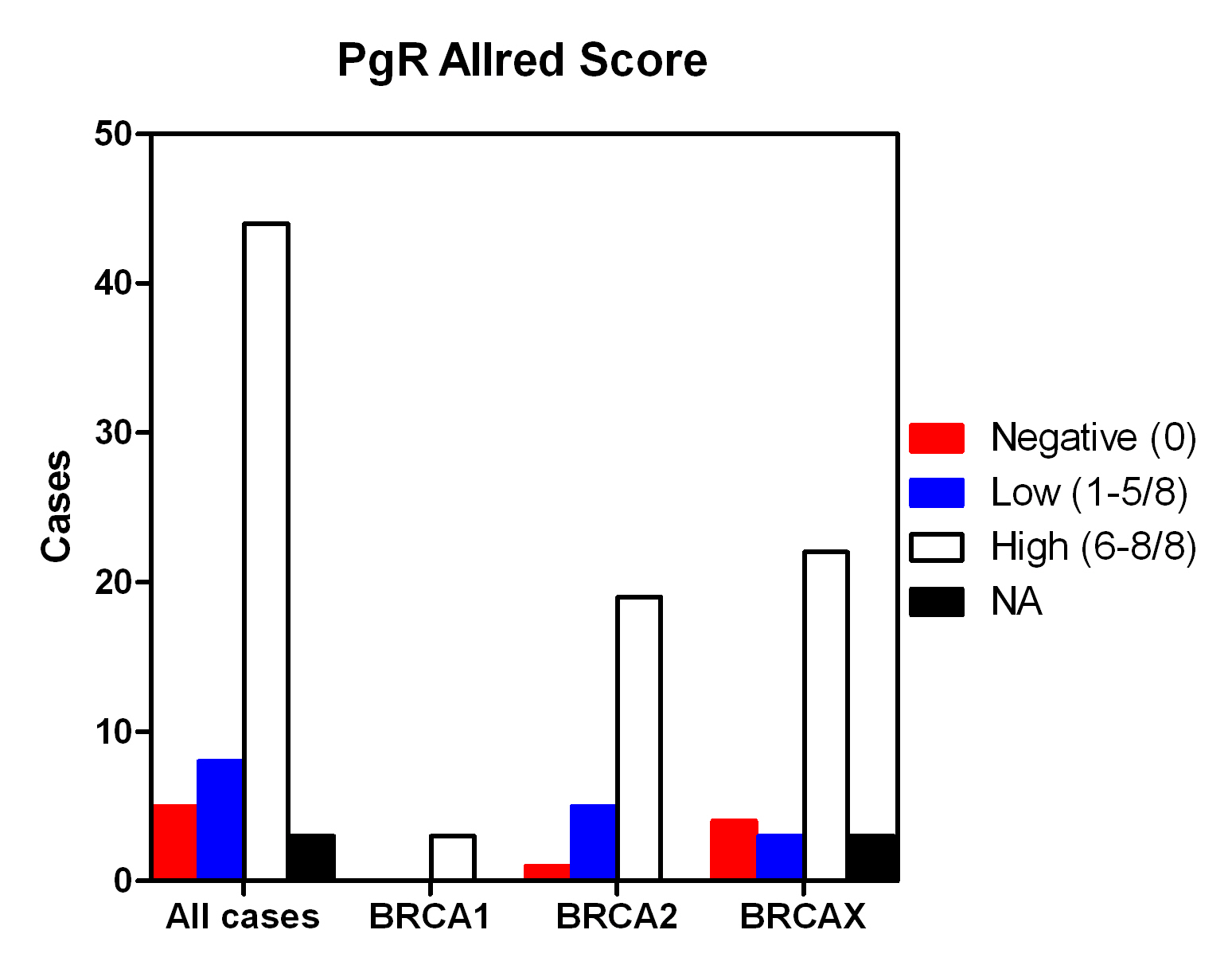

Supplement: Additional file 3 — Figure S2. Distribution of PgR Allred histoscores. [file 1471-2407-12-510-S3.jpeg]
